# OsCBE1, a Substrate Receptor of Cullin4-Based E3 Ubiquitin Ligase, Functions as a Regulator of Abiotic Stress Response and Productivity in Rice

**DOI:** 10.3390/ijms22052487

**Published:** 2021-03-02

**Authors:** Juyoung Choi, Wonkyung Lee, Gynheung An, Seong-Ryong Kim

**Affiliations:** 1 Department of Life Science, Sogang University, Seoul 04107, Korea; cjy1835@naver.com (J.C.); wk971018@naver.com (W.L.); 2 Department of Plant Molecular Systems Biotechnology, Kyung Hee University, Yongin 17104, Korea; genean@khu.ac.kr

**Keywords:** abiotic stress, cullin4, crop productivity, E3 ubiquitin ligase, rice

## Abstract

Ubiquitination is an important environmental stress response, and E3 ubiquitin ligases play a major role in the process. T-DNA insertion mutants of rice, *Oscbe1-1*, and *Oscbe1-2,* were identified through the screening of cold stress tolerance at seedling stage. *O*s*cbe1* mutants showed a significantly higher cold stress tolerance in the fresh weight, chlorophyll content, and photosynthetic efficiency than wild type. Molecular prediction showed that *OsCBE1* (*Oryza sativa* Cullin4-Based E3 ubiquitin ligase1) encoded a novel substrate receptor of Cullin4-based E3 ubiquitin ligase complex (C4E3). Whereas *Oscbe1* mutants had fewer panicles and grains than wild type in the paddy field, the overexpression lines of *OsCBE1* had more panicles and grains, suggesting that *OsCBE1* is involved in the regulation of both abiotic stress response and development. *Oscbe1* mutants also showed ABA hypersensitivity during seed germination, suggesting *OsCBE1* function for the stress response via ABA signaling. In silico analysis of OsCBE1 activity predicted a CCCH-type transcription factor, OsC3H32, as a putative substrate. Co-IP (Co-immunoprecipitation) study showed that OsCBE1 interacts with OsDDB1, an expected binding component of OsCBE1 and OsC3H32. Additionally, expression of *OsOLE16, OsOLE18*, and *OsBURP5* were negatively related with expression of *OsCBE1*. These results suggest that *OsCBE1* functions as a regulator of the abiotic stress response via CCCH as a member of the C4E3.

## 1. Introduction

Since plants are sessile, it is hard for a plant to escape from continually changing environments. These external conditions are often unfavorable and the environmental conditions strictly limit geographical distribution of plants and productivity in agriculture [1]. The productivity of crops decreases more than 50% from abiotic stress [2], and global climate change is threatening plant habitats [3]. Therefore, the understanding of the abiotic stress response of plants is necessary to meet the global food demand under global climate change.

In stress condition, there are various changes in plants, such as gene expressions and metabolite composition [4]. To make these changes, the stress sensing-signaling process is required, and a few putative stress sensors such as *OSCA1* [5] and *COLD1* [6] have been reported. In the stress signaling, phytohormone abscisic acid (ABA) is one of the most important signaling molecules. Binding of ABA to PYR/PYL/RCAR families [7] and PP2Cs [8] evokes signaling cascades regulating PP2Cs [8] and SnRKs [9]. Then, at the end of the signaling cascades, various transcription factors of the bZIP [10], MYB/MYC [11], CBF/DREB [12], and NAC [13] families regulate expression of stress-responsive genes. Some CBF/DREB [14,15] and MYB [16] family members are regulated by ABA-independent signaling pathway. Consequently, physiological and molecular changes occur to adapt and survive in harsh conditions.

Post-translational modifications of proteins, such as phosphorylation and ubiquitination, have a vital role in the signal transduction of abiotic stress response as well [17]. Ubiquitination regulates various biological functions in plants. Ubiquitination occurs with three steps: 1. ATP dependent activation of ubiquitin by E1 ubiquitin-activating enzyme. 2. Transfer of ubiquitin to E2 ubiquitin-conjugating enzyme. 3. Transfer of ubiquitin to target and forming covalent bonds between lysin residues in ubiquitin and target by E3 ubiquitin ligase [18]. There are various kinds of ubiquitination, including mono- or poly-ubiquitination, and each ubiquitination may cause different kinds of regulation, such as protein degradation by 26S proteasome, activation, and localization [19].

In the plant species, rice has 1483 putative E3 ubiquitin ligase genes (4.3% of protein-coding genes in rice), and *Arabidopsis* has 1424 putative E3 ubiquitin ligase genes (5.4% of protein-coding genes in *Arabidopsis*) [20,21,22,23]. Monomeric E3 ubiquitin ligases of HECT, U-box, and RING work alone [24]. Meanwhile, multimeric E3 ubiquitin ligases of SCF complex [25], APC/C- [26], cullin 3- [27], and cullin 4- [23], work with cullin or other components as complexes. Since the cullin 4-based E3 ubiquitin ligase complex (C4E3) was first reported in *Arabidopsis* [28], various functions of C4E3 have been elucidated, including regulation of photomorphogenesis [28], embryogenesis [29], flowering [30], immunity [31], and stress signaling [32]. The C4E3 consists of cullin 4, RBX1, DDB1, and a substrate receptor named DCAF (DDB1 Cullin4 Associated Factor) [33]. DCAFs commonly have a conserved 16-17 amino acid motif called DWD box (DDB1 binding WD40), which gives a binding site for DDB1 [29]. DCAFs containing only WDxR motifs, which are 13–16th residues of the DWD box, have also been reported as non-DWD DCAFs, where WDxR is crucial for the binding of the DCAFs to DDB1 [34]. It has been estimated that there are 151 and 119 DCAFs in rice and *Arabidopsis*, respectively [22,23], although most of them remain to be characterized.

In this study, we studied an abiotic stress tolerant mutant, *Oscbe1*, and characterized it at the molecular level. OsCBE1, a putative novel DCAF protein, was further characterized for its physiological role in the regulation of abiotic stress tolerance and crop productivity. Furthermore, an in silico identification of OsCBE1 substrate, OsC3H32, was further characterized in detail.

## 2. Results

### 2.1. Identification of Cold Stress Mutant Oscbe1

**Figure 1:**
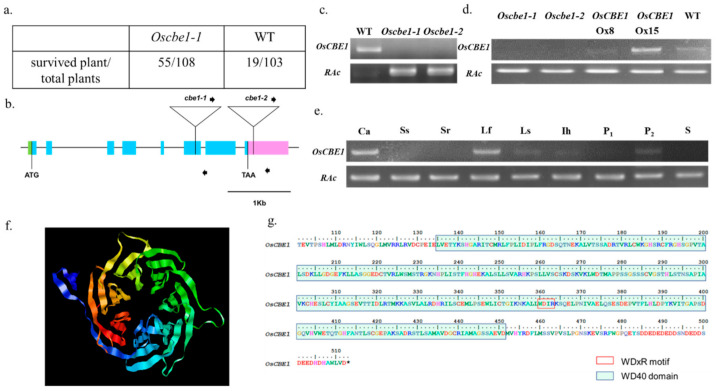
Screening of Cold tolerant E3 ubiquitin ligase line. (**a**) Survival rates of 7d rice seedlings after low temperature treatment. (**b**) Map depicting the tagging position of two allelic T-DNA insertion mutant lines of *OsCBE1*. Exons (blue boxes), 5′UTR (green box), 3′UTR (pink box), and introns (lines) are indicated. (**c**) Identification of *Oscbe1* T-DNA insertion mutants (**d**) Expression profile of *OsCBE1* in mutant and overexpression lines. (**e**) Expression profile of *OsCBE1* in different tissues of rice. Ca, callus; Ss, shoots at 7 days after germination (DAG); Sr, roots at 7 DAG; Lf, mature leaves; Ls, flag leaf sheaths; Ih, the highest internode at pre-heading stage; P1, 1–2 cm panicles; P2, 3–8 cm panicles. (**f**) Predicted seven bladed beta propeller structure of WD domains in OsCBE1 protein. Image colored by rainbow N-to C-terminus. (**g**) WD domain of OsCBE1 with WDxR motif.

To investigate the function of E3 ubiquitin ligase on the abiotic stress response, we have screened a mixed pool of transgenic rice with T-DNA tagged mutation of putative E3 ubiquitin ligase genes for the cold stress tolerance. A cold tolerant mutant *Oscbe1-1* (PFG_4A-50680) was identified (Figure 1a), and the T-DNA tagging loci (LOC_Os01g09020) was confirmed by inverse PCR. The corresponding gene was named *Oryza sativa* Cullin4-Based E3 ubiquitin ligase1 (*OsCBE1*). An allelic mutant line (*Oscbe1-2*, PFG_4A-01910) of *Oscbe1-1* was identified from RiceGE (http://signal.salk.edu/cgi-bin/RiceGE), and the T-DNA insertion loci was confirmed on the 3′-UTR of *CBE1* (Figure 1b). From the T2 generation of both *Oscbe1* mutants, homozygote T-DNA tagging mutants were identified by genotyping, as shown in Figure 1c, and were confirmed via checking the expression of *OsCBE1* (Figure 1d). In addition, *OsCBE1* overexpression (Ox) lines were made to study the function of the gene. Of several Ox lines obtained, Ox8 and Ox15 were selected and further examined with the *Oscbe1* mutants. As shown in Figure 1d, *OsCBE1* Ox8 and Ox15 showed 1.23 and 5.55 times stronger expression of *OsCBE1*, compared to the wild type, respectively. The expression of the *OsCBE1* gene during development was examined in various tissues by RT-PCR. As shown in Figure 1e, *OsCBE1* was highly expressed in the callus and mature leaves, but barely detected in the seedlings. *OsCBE1* contains 1539 bp of the open reading frame and encodes 512 amino acid-long polypeptides. Using the NCBI conserved domain search tool [35], seven WD repeat domains were found in the OsCBE1. These domains were predicted to have a seven bladed β-sheet propeller-like structure (Figure 1f) using Robetta server [36]. Even though DWD box was not detected in *OsCBE1*, one of the seven WD domains of *OsCBE1* at the position of amino acid 360 showed a WDxR motif (Figure 1g). These indicate that *OsCBE1* has conserved sequences of DCAF and may work as a non-DWD DCAF, where WDxR is crucial for the binding of the DCAF to DDB1.

### 2.2. OsCBE1 Negatively Regulates Stress Response and ABA Signaling

**Figure 2:**
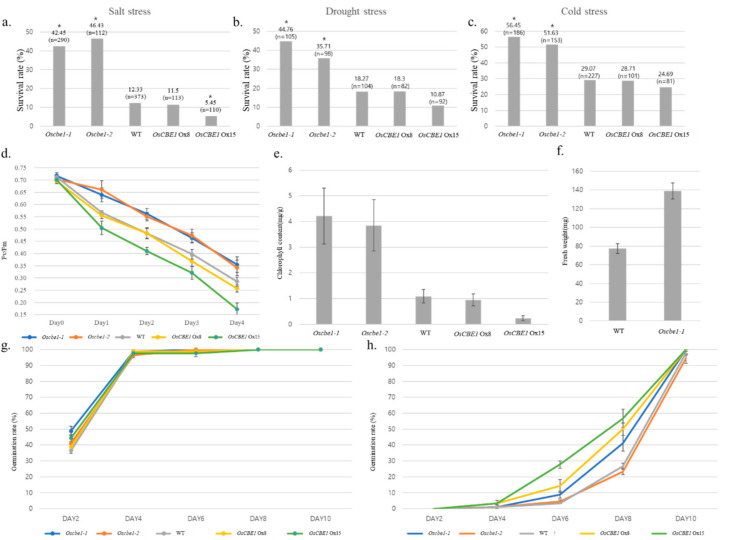
Phylogenetic traits of *CBE1* lines. Abiotic stress tolerance was analyzed with survival rate in *OsCBE1* lines. Before stress treatment, each plant was grown in soil for seven days. (**a**) Salt stress was treated for 84 h by irrigating with 250 mM NaCl solution. Survival rate was analyzed, after 7 days of recovery. (**b**) Drought stress treatment for 36 h by withholding water. Survival rate was analyzed after seven days of recovery. (**c**) Cold stress treatment for 96 h under 4 °C and 130 μMm^−2^s^−1^ light intensity. Survival rate was analyzed after seven days of recovery. (**d**) Decrease of chlorophyll florescence (Fv/Fm) of *OsCBE1* lines during cold stress in 4 °C and 130 μMm^−2^s^−1^ light intensity was analyzed. (**e**) Chlorophyll contents of *OsCBE1* lines after cold stress in 4 °C and 130 μMm^−2^s^−1^ light intensity for four days were analyzed. (**f**) Fresh weights of *OsCBE1* lines after salt stress in 250 mM NaCl treatment for 84 h were analyzed. (**g**) Germination rate of transgenic lines of *OsCBE1* were analyzed in a ½ MS, 27 °C, dark condition after 50% bleach treatment for 40 min. (**h**) Germination rate of transgenic lines of *OsCBE1* were analyzed in ½ MS, 5μM ABA, 27 °C, dark condition after 50% bleach treatment for 40 min. *, significant difference from wild type (Chi-square test with *p* < 0.05); Error bar, ± Standard error.

To investigate the role of *OsCBE1* in abiotic stress, various stress tolerance of *Oscbe1* mutants with Ox lines were estimated as survival rates after the recovery from the stress of salt, drought, and cold. For salt stress, the survival rates of both *Oscbe1-1* and *Oscbe1-2* were more than 40%, whereas the rate was 12.3%, 11.5%, and 5.5% for the wild type, *OsCBE1* Ox8 (1.23 times overexpressed than wildtype), and *OsCBE1* Ox15 (5.55 times overexpressed than wildtype), respectively (Figure 2a). For drought stress, more than 35% of both *Oscbe1-1* and *Oscbe1-2* seedlings survived, whereas the rate was 18.3%, 18.3%, and 10.9% for the wild type, Ox8, and Ox15, respectively (Figure 2b). For cold stress, the survival rates of both *Oscbe1-1* and *Oscbe1-2* were more than 50%, whereas those of the wild type and Ox lines were less than 30% (Figure 2c). For the tested abiotic stresses, both *Oscbe1-1* and *Oscbe1-2* showed significantly higher survival rate than that of the wild type, as estimated by Chi-square analysis with *p* < 0.05. Whereas *OsCBE1* Ox8 showed similar survival rate to wild type, the *OsCBE1* Ox15 line showed significantly lower survival rate to salt stress and slightly lower survival rate to cold and drought stress. During the cold stress treatment up to four days, Fv/Fm, an indicator of the quantum yield of photosystem II, was rather slowly decreased in *Oscbe1* mutants, compared to either wild type or *OsCBE1* Ox lines. While Fv/Fm of *Oscbe1-1* and *Oscbe1-2* were 0.35 and 0.34, respectively, the value of *OsCBE1* Ox15 declined to 0.17 after four days of cold treatment (Figure 2d). After seven days of recovery from the cold stress treatment, the chlorophyll contents of both *Oscbe1* mutants were about 4.21 ± 1.21 mg/g of fresh weight (FW), which was higher than that of the wild type (1.0 mg/g FW). *OsCBE1* Ox15 had a much lower chlorophyll content of 0.23 ± 0.07 mg/g FW, although, *OsCBE1* Ox8 had similar chlorophyll content to the wild type (Figure 2e). Regarding salt stress of 250 mM NaCl for 84 h, the average FW of *Oscbe1-1* after seven days of recovery was 139.1 ± 8.6 mg. Meanwhile, the value of the wild type was 77.4 ± 5.1 mg (Figure 2f). These physiological changes after abiotic stress treatment suggest that OsCBE1 work as a negative regulator of abiotic stress tolerance.

In addition, leaf disc assay was performed to analyze salt stress and cold stress tolerance of mature plants (≈60 DAG). Leaf disk assay, under various concentrations of NaCl, revealed that more chlorophyll remained in leaf discs of *Oscbe1-1* and *Oscbe1-2* than in the wild type (WT) plants (Figure S1a–c). After five days of salt stress treatment, the chlorophyll contents in leaf discs in *Oscbe1-1* and *Oscbe1-2* were about 4.8 ± 1.12 mg/g of FW and 1.06 ± 0.256 mg/g of FW, which were higher than that of the wild type (0.9 ± 0.093 mg/g FW). Leaf discs of *OsCBE1* Ox15 had a much lower chlorophyll content of 0.36 ± 0.123 mg/g FW (Figure S1c). Additionally, Fv/Fm slowly decreased in leaf disks of *Oscbe1* mutants, compared to leaf disks of the wild type or *OsCBE1* Ox lines. After five days of floating on autoclaved water at 4 °C, Fv/Fm of leaf discs of *Oscbe1-1* and *Oscbe1-2* were 0.42 and 0.372, respectively. However, leaf discs of wild type and *OsCBE1* Ox8 had lower Fv/Fm of 0.195 and 0.190, respectively. Additionally, leaf discs of *OsCBE1* Ox15 had a much lower Fv/Fm of 0.097 (Figure S1d).

To analyze whether OsCBE1 was related to the ABA signaling pathway, we germinated seeds of *Oscbe1* mutants and Ox lines in agar media containing ABA. In the media without ABA, germination rate was similar between the wild type and *OsCBE1* lines (Figure 2g). In the media with 5 μM ABA, germination delay was different in each line. Whereas *Oscbe1* mutants showed hypersensitivity to ABA during germination, the Ox lines showed slightly enhanced germination compared to either the wild type or *Oscbe1* mutants (Figure 2h).

### 2.3. Expression of OsCBE1 Positively Related to Crop Productivity

**Figure 3:**
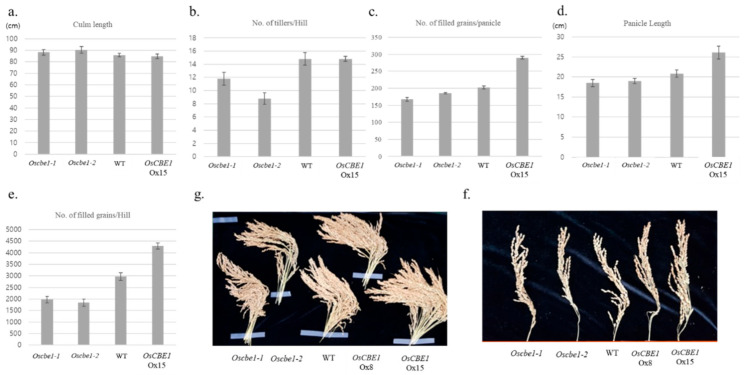
Agronomical traits of *CBE1* lines. (**a**–**e**) Agronomical traits of *OsCBE1* lines are presented. (**a**) Culm length; (**b**) Number of tillers per hill; (**c**) Number of filled grains per panicle; (**d**) Panicle Length; (**e**) Number of filled grains per hill. Each of the results are presented as means ± standard error. (**f**) Panicles from a single *OsCBE1* line plant are presented. (**g**) Single panicle of each *OsCBE1* line are presented.

The knockout mutants, *Oscbe1-1* and *Oscbe1-2*, and the overexpression line Ox15 were grown in a paddy field in the normal growth condition. Culm length were similar between those mutants and the wild type (Figure 3a). However, *Oscbe1* mutants had less tillers than wild type and Ox15. The number of tillers per plant were 9–12 from *Oscbe1* mutants, versus 15 from wild type and Ox15 (Figure 3b,g). Whereas the number of filled grains per panicle from *Oscbe1* mutants were 10–20% less than those from wild type, Ox15 had over 40% more filled grains per panicle than wild type (Figure 3c,f). Similarly, compared to wild type, the length of panicles was ≈10% shorter in *Oscbe1* mutants and ≈30% longer in Ox15 (Figure 3d,f). Therefore, the grain yield per plant of *Oscbe1* mutants and Ox15 were 34–38% less and 45% more, respectively, than that of the wild type. (Figure 3e,g). Taken together, the expression of *OsCBE1* shows a positive relation to agronomical productivity in rice.

### 2.4. Functional Role of OsCBE1 and In Silico Identification of the OsCBE1 Binding Partners

**Figure 4:**
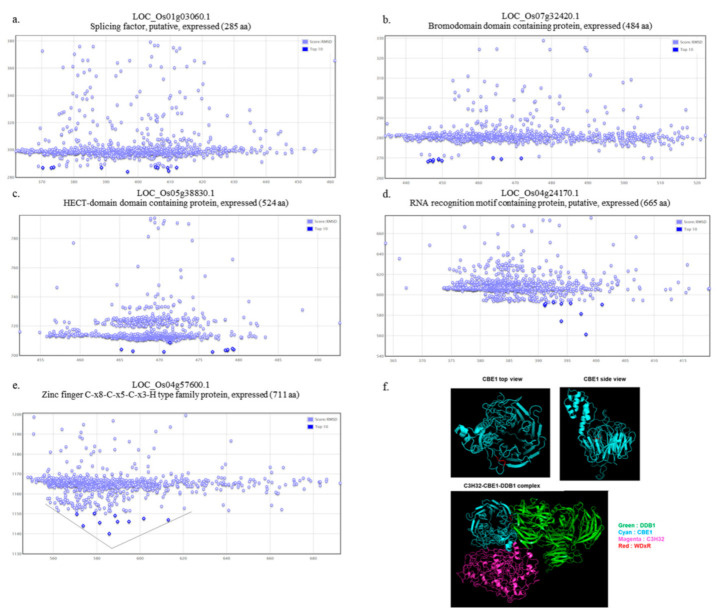
In silico identification of the OsCBE1 binding partners. (**a**–**e**) Energy plot of interaction energy between OsCBE1 and five putative substrates, LOC_Os01g03060.1 (**a**), LOC_Os07g32420.1 (**b**), LOC_Os05g38830.1 (**c**), LOC_Os04g24170.1 (**d**), and LOC_Os04g57600.1 (**e**), and Root-mean-square deviation of atomic positions (RMSD). Energy diagram of LOC_Os01g03060.1, LOC_Os07g32420.1, and LOC_Os05g38830.1 have horizontal distribution. Energy diagram of LOC_Os04g24170.1 has three downward plots, but most of the distribution is horizontal. Only the energy diagram of LOC_Os04g57600.1 shows funnel-like distribution (marked with black lines). (**f**) Predicted structure of OsCBE1 and OsDDB1-OsCBE1-OsC3H32 complex.

Since *OsCBE1* has conserved sequences of substrate receptor of C4E3, putative interaction partner of OsCBE1 was examined in silico with biological evidence and protein docking simulation. From the STRING database [37], five candidate genes were identified based on co-expression, co-occurrence, and evidence of biological relation between genes (Figure S2, Table S1), although there was no experimental evidence. Therefore, interaction modeling with OsCBE1 was performed using both Phyre2.0 server and ROSIE server docking2 protocol for further analysis [38,39]. The energy distribution analysis from the docking simulation with only one candidate, OsC3H32, showed a funnel-shaped distribution (Figure 4a–e). Since DCAFs in C4E3 interact with both substrate and DDB1, the structure of the C4E3 complex was predicted in silico as well. The predicted structure of OsCBE1 looks like a “ladle” with a “bowl” composed of a seven-bladed β-propeller and “handle” composed of several α-helixes. A simulated complex with the lowest energy showed that OsC3H32 binds to the “handle” of OsCBE1. Since the WDxR motif is placed in the “bowl” of OsCBE1, the predicted complex, OsDDB1-OsCBE1-OsC3H32, formed without conformational hindrance (Figure 4f). Therefore, OsC3H32 was selected as a putative substrate of OsCBE1 and further characterized.

### 2.5. Physical Interaction of OsCBE1 with OsDDB1 and OsC3H32

**Figure 5:**
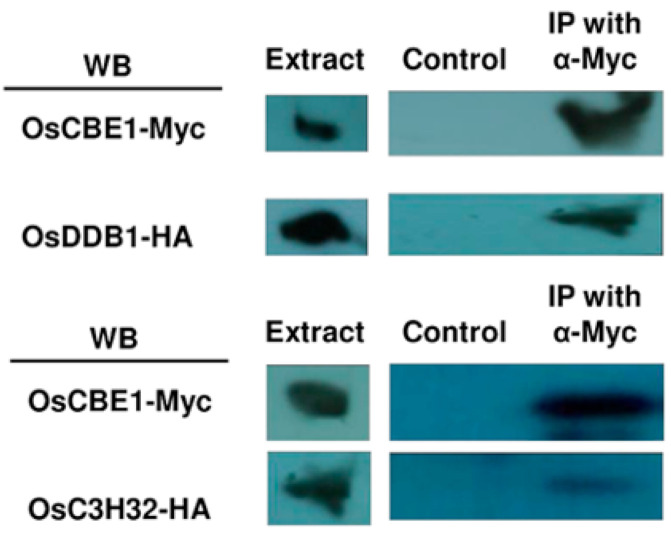
Co-IP with OsCBE1 and predicted substrate. To confirm whether the putative substrate from the in silico method can interact with OsCBE1, we performed Co-IP using transient expressed Myc tagged OsCBE1 and HA tagged predicted substrate in rice suspension cell.

In order to examine whether OsCBE1 was a substrate receptor of C4E3, the binding of OsCBE1 with OsDDB1 was examined. Both expression vectors of Myc-tagged OsCBE1 and HA-tagged OsDDB1 were constructed using pGA3817 and pGA3818, respectively. The vectors were co-transformed by electroporation into rice protoplasts prepared from the suspension cells of Dongjin wild type. Protein extracts from the transformed cells were used for Co-IP (Co-immunoprecipitation) analysis (See Materials and Methods). As shown in Figure 5, the OsDDB1 of 120 kD was immunoprecipitated with the 61 kD OsCBE1. However, the HA tag, negative control, was not immunoprecipitated with the OsCBE1 (Figure S3). We further examined whether OsCBE1 interacted with OsC3H32, the in silico predicted substrate in this study. An expression vector of HA-tagged OsC3H32 was constructed using pGA3818 and then co-transformed into rice protoplast with the Myc-tagged OsCBE1 vector. The OsC3H32 of 77 kD was also immunoprecipitated with the OsCBE1 (Figure 5), indicating that OsC3H32 could be a substrate of OsCBE1.

### 2.6. Oleosin Genes and an ABA Responsive Gene Are Negatively Regulated by OsCBE1

**Figure 6:**
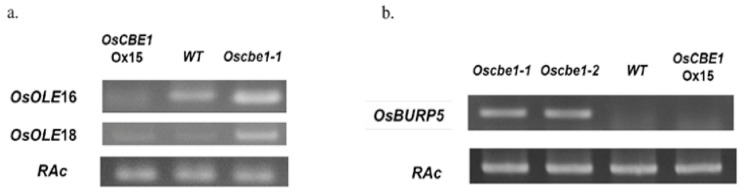
Regulation of *OsOLE16*, *OsOLE18,* and *OsBURP5* in *OsCBE1* lines. (**a**) Expression of *OsOLE16* and *OsOLE18* in *OsCBE1* lines are presented. *OsOLE16* and *OsOLE18* are up-regulated in KO line. *OsOLE16* are down-regulated in the overexpression line. (**b**) Expression of *OsBURP5* in *OsCBE1* lines are presented. *OsBURP5* is upregulated in KO lines.

Based on the fact that OsC3H32 could be a substrate of OsCBE1, the function of the OsC3H32 was studied in the literature. Since the function of *OsC3H32* has not been reported, an *Arabidopsis* homologue was searched using BLAST. It was reported that *AtC3H17* is a homologue of *OsC3H32* with an amino acid identity of 35.4% [40]. *AtC3H17* was reported to upregulate oleosin genes, *AtOLEO*1 and *AtOLEO*2, which had a pleiotropic function in development [41] and salt stress tolerance via ABA signaling pathway [40]. Therefore, the corresponding homologues of *AtOLEO*1 and *AtOLEO*2 were found in rice first. *OsOLE*16 and *OsOLE*18 were obtained as homologues of *AtOLEO*1 and *AtOLEO*2, respectively. Then, the expression of *OsOLE*16 and *OsOLE*18 were analyzed in the *Oscbe1* and *OsCBE1*-Ox lines by qRT-PCR. As shown in Figure 6a, whereas the expression of *OsOLE*16 and *OsOLE*18 were increased in *Oscbe1-1*, the expression of *OsOLE*16 was decreased in *OsCBE1-Ox15*. The expression of *OsOLE*18 in the Ox line was similar to the wild type. We also examined the expression of *OsBURP5*, a rice homologue of the well-known ABA responsive gene *AtRD22.* The *OsBURP5* mRNA was increased in *Oscbe1* mutants, but barely detected in the Ox line, indicating *OsCBE1* might positively regulate *OsBURP5* in rice (Figure 6b).

## 3. Discussion

In this study, we showed abiotic stress tolerance of *Oscbe1* and the stress sensitivity of *OsCBE1*-*Ox15,* with various stress markers such as survival rates and chlorophyll fluorescence. ABA responses, germination delay, and ABA responsive gene expression were increased in *Oscbe1* and were decreased in *OsCBE1-Ox15*, suggesting that OsCBE1 negatively regulates abiotic stress response through at least part of ABA pathway.

OsCBE1 belongs to non-DWD DCAFs, which was first shown in rice. It has been reported that DCAFs of ABD1 [42], ASG2 [43], DET1-DDA1 complex [44,45], DRS1 [46] DWA1, DWA2 [32], DWA3 [47], HOS15 [48], RAE1 [49], and WDR55 [50] regulate the ABA signaling pathway. With ABD1, OsCBE1 is another non-DWD type DCAF protein related to the ABA pathway. Recently, it was shown that a rice cullin4 gene, *OsCUL4*, was also induced by ABA, drought, and salt stress [51].

Most DCAFs, including OsCBE1, negatively regulates the ABA pathway, except DRS1 and WDR55 [46,50]. The functional regulation of DCAFs on the ABA pathway seems due to the character of C4E3. DDA1, the substrate receptor of C4E3, also negatively regulates the ABA pathway itself [52]. Whether the negative regulation on ABA pathway by DDA1 is related to the structural stabilization of C4E3 by interacting with DCAFs needs further studies. Similarly, most U-box E3 ubiquitin ligases negatively regulate the ABA pathway [53,54,55,56,57,58,59,60]. Contrary to C4E3, many RING-type E3 ubiquitin ligases positively regulate the ABA pathway [56,60]. Therefore, this bias of E3 ubiquitin ligases on ABA regulation pathway would be tuned and established during molecular evolution of this enzyme.

Photosynthetic efficiency is one of the major metabolic factors for increasing biomass. More than 90% of crop biomass is derived from photosynthetic products [61]. Although our *Oscbe1* mutants showed overall abiotic stress tolerance (Figure 2), they showed a reduction in crop productivity in the normal paddy field (Figure 3). In many cases, rice production is limited by grain number [62,63]. *Oscbe1* showed reduced tiller numbers and, therefore, reduced grain numbers compared to the wild type. It is remarkable that *OsCBE1-Ox15* showed high yield increase compared to the wild type. *OsCBE1-Ox15* showed increased production of agronomic traits, such as panicle length and grain numbers, than the wild type. We suggest that *OsCBE1* acts as a positive regulator for tiller formation and grain ripening. The stress tolerance and productivity were often reversed. Overexpression of *OsPYL/RCAR5,* an ABA receptor in rice, enhanced drought tolerance but severely reduced yields [64]. The knockout of the *OsPYL* family, which were drought sensitive, showed an increase in crop yield in normal conditions [65].

**Figure 7:**
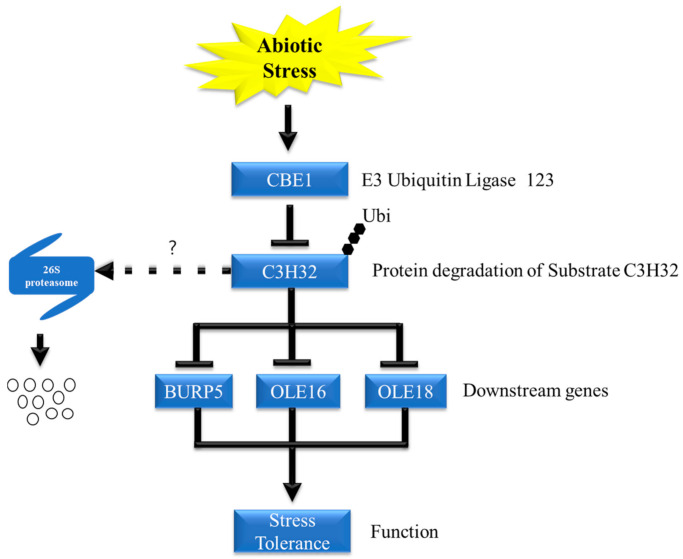
Model of OsCBE1 functioning in regulating abiotic stress response in rice. The arrow represents activation while the flat arrow represents inhibition. The dotted arrow and question mark represent hypothesis about how it works.

In this study, we found a substrate of OsCBE1 (Figure 4 and Figure 5). One of the reasons that only a few E3 ubiquitin ligases were functionally characterized is the difficulty in finding their substrates. Many tools, including yeast-2-hybrid screening, mass spectrometry-based proteomics, substrate trapping proteomics, global protein stability profiling, protein microarray, phage display, and high-throughput quantitative microscopy, have been used and developed for decades [66,67,68,69,70,71,72]. However, these are laborious and time-consuming. Additionally, there are many hurdles to identify substrate: 1. Too weak and short interactions between substrates and E3 ubiquitin ligases. 2. Delicate regulation of ubiquitination depends on various environments. 3. Rapid degradation and low cellular content of substrates [73]. These increase failure probability for finding out substrates and makes E3 ubiquitin ligase characterization difficult. In silico prediction tools, which are faster and less laborious, were successfully used in this study to find an interacting partner, OsC3H32, a CCCH family zinc finger protein. The in silico prediction was performed in three steps: 1. Interacting partner candidate sorted with biological evidences. 2. Protein docking simulation. 3. Docking funnel analysis and complex structure analysis. There are many available E3 ubiquitin ligases related OMICS database, including co-expression, co-occurrence, co-localization, protein interaction network and related phenotypes in transgenic lines, and E3 ubiquitin ligase-substrate interface database. In this study, five substrate candidates were sorted with co-expression and co-occurrence from the STRING database [37]. Recently, a new platform [74] which analyzes biological evidence by analyzing sequences and interactome databases was developed and would be helpful for sorting out substrate candidates. Co-IP data showed that OsCBE1 indeed binds to OsC3H32 in living rice cells (Figure 5). Although the function of OsC3H32 has not been characterized in rice, an *Arabidopsis* ortholog, AtC3H17, increased the expression levels of oleosin genes, *AtOLEO*1 and *AtOLEO*2 [41]. The expression levels of *OsOLE16* and *OsOLE18* were also increased in the *Oscbe1* mutants. Moreover, Seok et al. reported that expression level of the ABA responsive gene, *RD22* was increased by *AtC3H17* [40]. We similarly confirmed that the expression of *OsBURP5*, a homologue of *RD22*, was increased in the *Oscbe1* mutants (Figure 6). Furthermore, *OsOLE16* and *OsOLE18* are known as ABA responsive genes too [75]. These results suggest that OsCBE1 act as a positive regulator for oleosin synthesis, and a negative regulator for ABA signaling by inhibiting the function of OsC3H32 (Figure 7).

Rice OsCBE1 is induced by abiotic stress and then ubiquitinates and may destabilize its substrate OsC3H32, by promoting its degradation through the 26S proteasome. OsC3H32 are downregulated under abiotic stress, following which inhibition of downstream genes (*OsBURP5, OsOLE16,* and *OsOLE18*) were lifted which resulted in abiotic stress tolerance.

CCCH family zinc finger proteins are widely found in various eukaryotes [76,77,78,79,80]. In plants, many CCCH family transcription factors are related to ABA pathways [80,81,82]. In rice, *OsC3H47* was highly induced by various stresses and ABA treatment, but the transcripts level of *OsC3H32* was not highly changed (less than twofold) in those stress conditions [83]. Therefore, it is suggested that OsC3H32 might be regulated at post-translational level, and OsCBE1 would be a major regulator of OsC3H32.

## 4. Materials and Methods

### 4.1. Plant Growth and Stress Tolerance Test

Rice plants were grown as previously described with small modifications [84]. Briefly, rice seeds (*Oryza sativa* ssp. japonica cv. Dongjin) were sterilized in 0.05% Spotak solution overnight at RT with rocking. Then, seeds were transferred to the sterilized wet paper tower and germinated in the dark at 28 °C for 2 days. Germinated seedlings were transferred to soil and cultured in a walk-in growth chamber (Koencon, Hanam, Korea) with 16 h of light (28 °C, 70% RH, 50 µmol/m^2^·s of light intensity) and 8 h of dark (22 °C, 70% RH).

For stress tolerance test for seedlings, cold, salt, and drought stress were treated as previously described with small modifications [84,85,86]. Briefly, seedlings 7 days after germination (DAG) were used. These were cold stress treated in a low-temperature chamber (Koencon, Hanam, Korea) with 16 h of daytime condition (4 °C, 60% RH, and 50 µmol/m^2^·s of light intensity) and 8 h of nighttime condition (4 °C, 60% RH, and 0 µmol/m^2^·s of light intensity); and salt stress treated in 250 mM of NaCl solution. Both salt stress and drought stress were treated in a growth chamber under the same conditions as the growth condition. Survivor rate was analyzed after seven days of recovery in the growth chamber.

To examine stress tolerance of mature plants, a leaf disc assay was conducted as previously described with small modifications [84]. Fully expanded leaves of healthy mature plants (≈60 DAG) were washed in deionized and autoclaved water. From these leaves, ≈8 cm diameter leaf disks were cut. For salt stress treatment, 100 mg of leaf discs were floated for 6 days in a 10 cm diameter petri dish filled with a 30 mL solution of various concentrations of NaCl (0, 250, and 500 mM). For cold stress treatment, 100 mg of leaf discs were floated in a 10 cm diameter petri dish filled with 30 mL of autoclaved water. Then, these were incubated for 5 days in a low-temperature chamber (Koencon, Hanam, Korea) with 16 h of daytime condition (4 °C, 60%RH, and 50 µmol/m^2^·s of light intensity) and 8 h of nighttime condition (4 °C, 60% RH, and 0 µmol/m^2^·s of light intensity).

### 4.2. Analysis of Chlorophyll Content and Fluorescence

The third leaf from each cold treated rice seedling adapted in the dark for 40 min. After dark-adaptation, chlorophyll fluorescence was measured with a Plant Efficiency Analyzer (Hansatech, King’s Lynn, UK). After stress treatment and 7 days of recovery, chlorophyll from each sample was extracted and quantified, as described previously [87].

### 4.3. PCR and RT-PCR Analysis

For genomic DNA extraction, 100 mg of young leaves from the seedling were collected and ground in an MM300 Mixer Mill (Retsch, Haan, Germany). DNA was extracted using the method of Chen and Roland [88] and the genomic DNA was amplified by PCR, as described previously [89].

Total RNA was extracted using an RNA Plants Kit (Machery-Nagel, Düren, Germany) from 200 mg of young leaves from the seedling collected. cDNA was synthesized from extracted RNA using TOPscript™ cDNA Synthesis Kit (Enzynomics, Daejeon, South Korea) and amplified for RT-PCR, as described previously [84]. Primer information for PCR and qRT-PCR analysis is in Table S2. Expression level of *OsCBE1* was analyzed with ImageJ [90].

### 4.4. Vector Construction

To construct *OsCBE1*-overexpression vector, a full-length open reading frame of *OsCBE1* was amplified from cDNA using Phusion High-Fidelity DNA Polymerase (Thermo Fisher Scientific, Waltham, MA, USA) with CBE1_*Mlu*1-F and CBE1_*Hin*dIII-R primer (Table S2). The amplified cDNA was introduced between the *Mlu*I and *Hin*dIII sites of pGA3436 binary vector under a maize ubiquitin promoter [91]. For the construction of Myc-tagged OsCBE1 expression vector, the full-length open reading frame of *OsCBE1* amplified with CBE1_*Hin*dIII-F and CBE1_*Kpn*I-R primer was inserted between the *Hin*dIII and *Kpn*I sites of pGA3817 vector [92]. For the construction of HA-tagged OsDDB1 vector and HA-tagged OsC3H32 vector, each ORF amplified with their respective primer (Table S2) was inserted into pGA3818 [92].

### 4.5. Generation of OsCBE1 Overexpression Lines

*OsCBE1* overexpression vector was transformed to calli of ‘Dongjin’ rice by *Agrobacterium*-mediated transformation methods, as described previously [84]. The transgenic plants were transferred to a paddy field in Kyung-Hee University (Suwon, Korea) for further growth.

### 4.6. Germination Test

To examine ABA sensitivity, rice seeds were germinated as described previously^41^ with small modifications [93]. Briefly, the 100 seeds per line were surface sterilized for 40 min and then transferred to ½ MS media, supplemented with 5 μM ABA, and germinated in the dark at 28 °C. Germinated seeds were counted every 12 h.

### 4.7. Evaluation of Agronomic Traits

Rice plants were grown at the LMO paddy field of Kyung-Hee University, Suwon, South Korea (permission number: RDA-가A-2011-039) from May to October. Five plants from each line were evaluated for agronomic traits, including the numbers of tillers, panicles, filled grain per plant, and the number of filled grain per panicle. Additionally, we measured lengths of the panicles of each plant.

### 4.8. In Silico Analysis

Co-expression, co-occurance data, and substrate candidate were retrieved from the STRING database with low criteria option [37]. Protein structure was predicted using the PHYRE 2.0 server with intensive mode protocol [39]. In silico docking was performed using the ROSIE server with merged PDB file [38,94]. To interpret docking results, IRMS (Interface Root-Mean-Square Deviation) of the docked complex was calculated with dockQ, and interaction energy was calculated with Rosetta3 [95,96]. The OsDDB1-OsCBE1-OsC3H32 complex structure was predicted using ZDOCK, and the WDxR motif of was OsCBE1 set as a binding site between OsCBE1 and OsDDB1 [97]. Every protein structure in this paper was illustrated using PYMOL [98].

### 4.9. Co-IP Analysis

For protein interaction analysis, Myc-tagged *OsCBE1* vector and HA-tagged *OsDDB1* or *OsC3H32* vector were co-transformed into rice calli using electroporation methods, as described previously [99]. After 16h from transfection, transfected cells were collected and Co-IP was performed, as described previously [100]. Briefly, 1 μL of anti-Myc antibody (Cell Signaling, 9B11), 10 μL protein A beads (Thermo Fisher Scientific, Waltham, Massachusetts, United States), and 10 μL protein G beads (Thermo Fisher Scientific, Waltham, MA, USA) were mixed in 1 mL of binding buffer (50 mM Tris-HCl, 75 mM NaCl, 5 mM EDTA, 1 mM DTT, 0.1 M PMSF, and 1% Triton X-100) for 3 h using a tube rotator. To extract total protein, transfected cells were incubated in the IP buffer (binding buffer with 1 X protease inhibitor cocktail, Roche, Basel, Switzerland). The extracted protein mixtures were precleared with 10 μL of protein A/G beads mixture for 1 h. Then, supernatant mixed with the anti-Myc antibody-bound protein A/G beads was incubated for 5 h on the tube rotator. Then, the beads were collected and washed, and the bound proteins were eluted in 20 μL of IP buffer. After immunoprecipitation, a western blot was performed with horseradish peroxidase conjugated Myc-tag mouse mAb (9B11, Cell Signaling Technology, Beverly, MA, USA) and HRP conjugated HA-tag mouse mAb (6E2, Cell Signaling Technology, Beverly, MA, USA).

## Data Availability

The data that support the results of this study will be provided from the corresponding author upon reasonable request.

## Supplementary Materials

Supplementary materials can be found at https://www.mdpi.com/1422-0067/22/5/2487/s1, Table S1: Information of primer sets for genotyping, vector construction and expression analysis, Table S2: Information of predicted substrates, selected by STRING score, Figure S1: Leaf disc assay of *OsCBE1* lines under salt and cold stress, Figure S2: Co-expression and Co-ocurrence data between *OsCBE1* and putative substrates, Figure S3: Negative control for *OsCBE1* binding assay.
