# Supplementary figures and images for: OsCBE1, a Substrate Receptor of Cullin4-Based E3 Ubiquitin Ligase, Functions as a Regulator of Abiotic Stress Response and Productivity in Rice
Source: Int J Mol Sci. 2021 Mar 2;22(5):2487. doi: 10.3390/ijms22052487 (PMC7957871; doi:10.3390/ijms22052487)

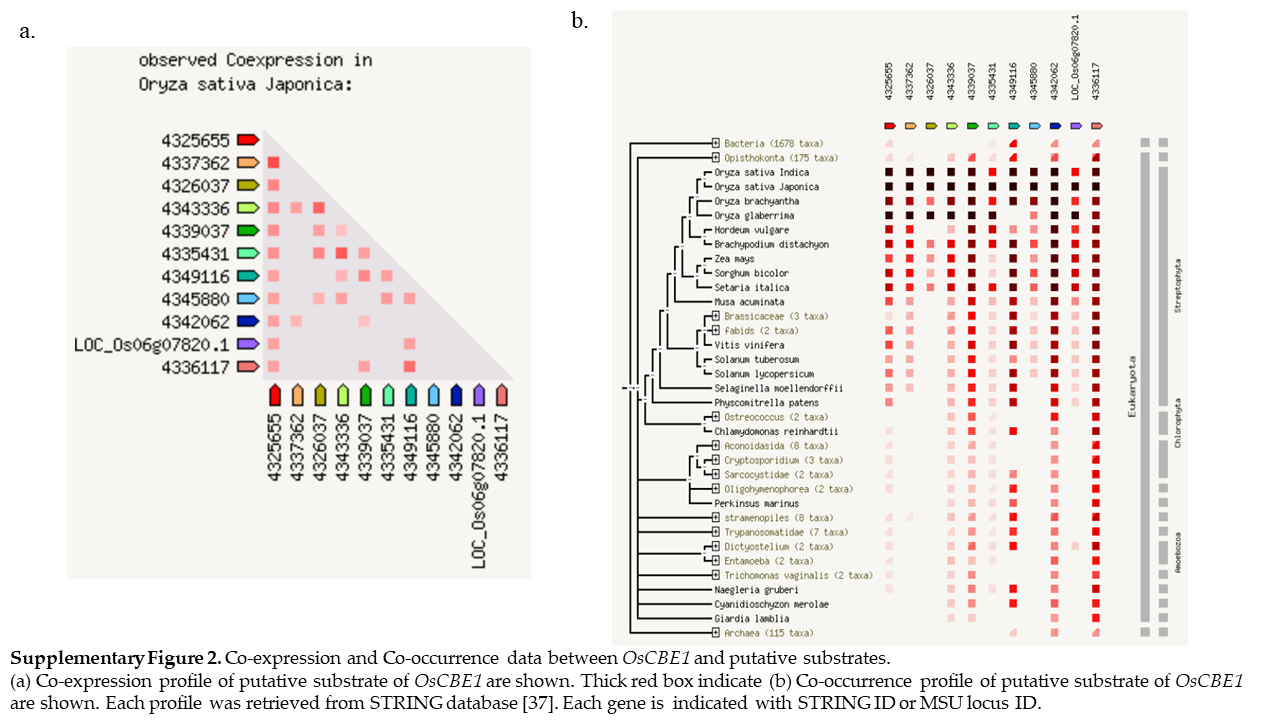

Supplement: Supplementary file 1 [file ijms-22-02487-s001.zip › 슬라이드10.TIF]

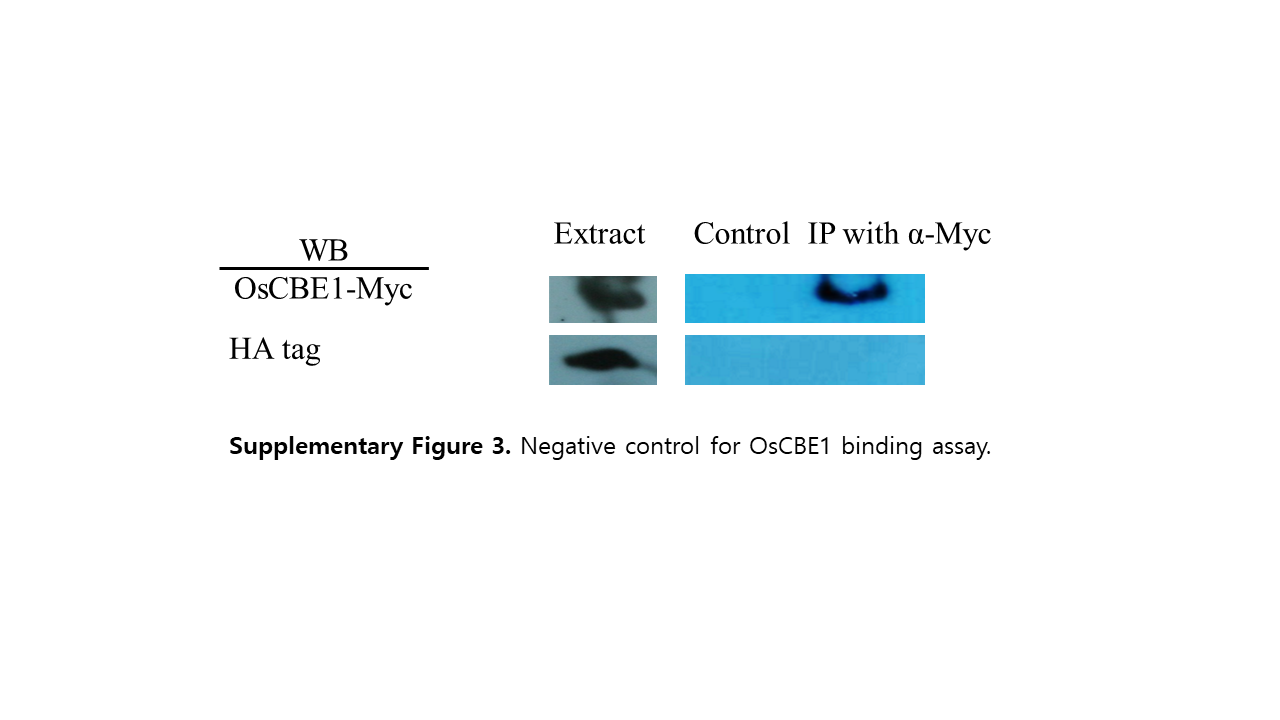

Supplement: Supplementary file 1 [file ijms-22-02487-s001.zip › 슬라이드11.TIF]

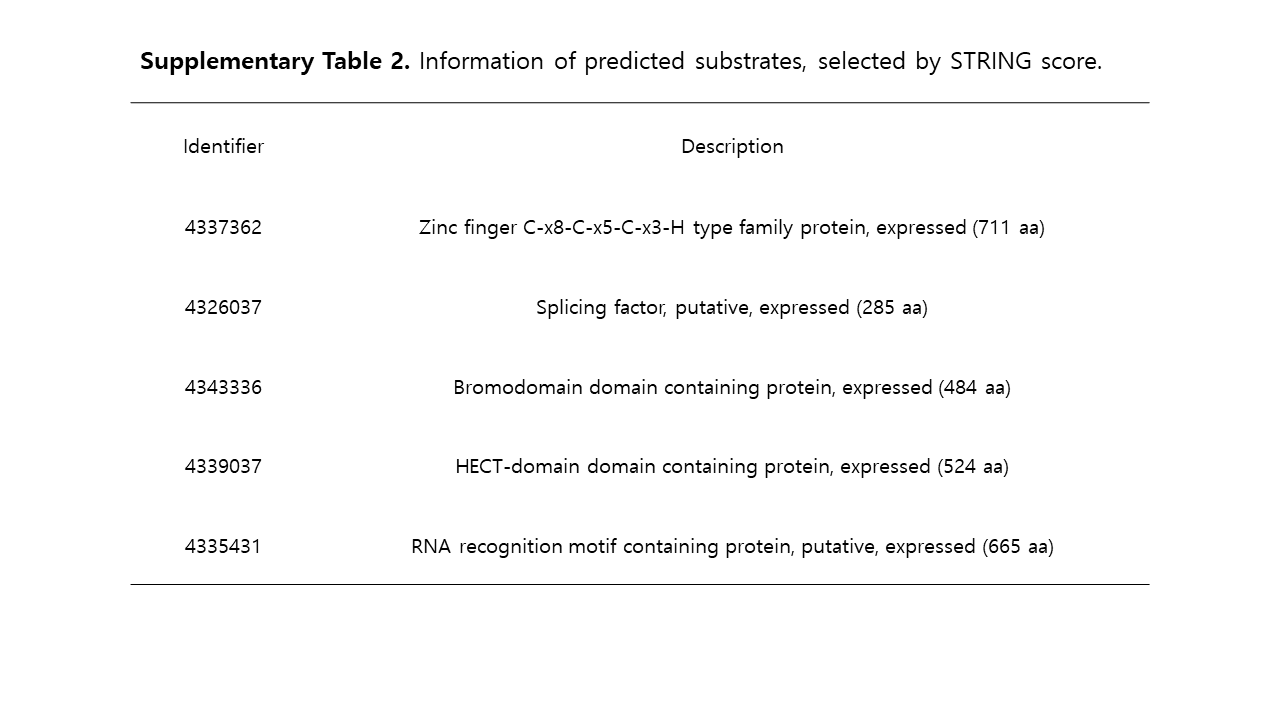

Supplement: Supplementary file 1 [file ijms-22-02487-s001.zip › 슬라이드12.TIF]

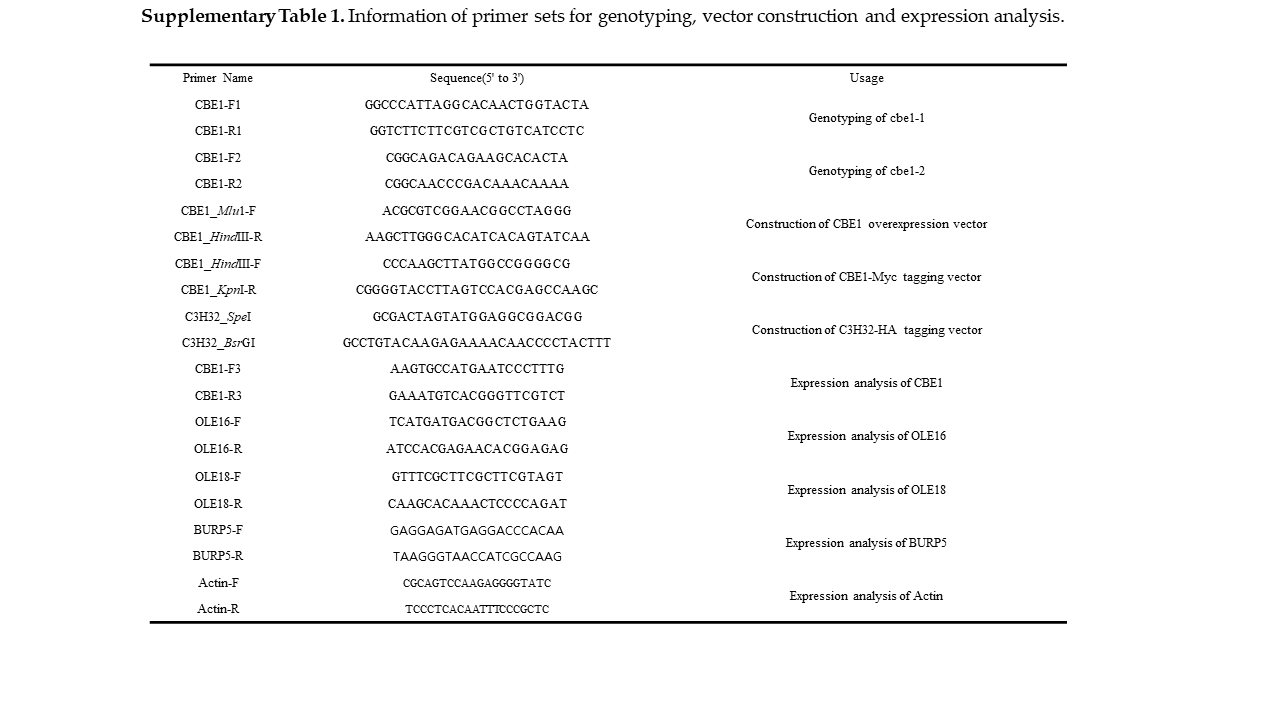

Supplement: Supplementary file 1 [file ijms-22-02487-s001.zip › 슬라이드8.TIF]

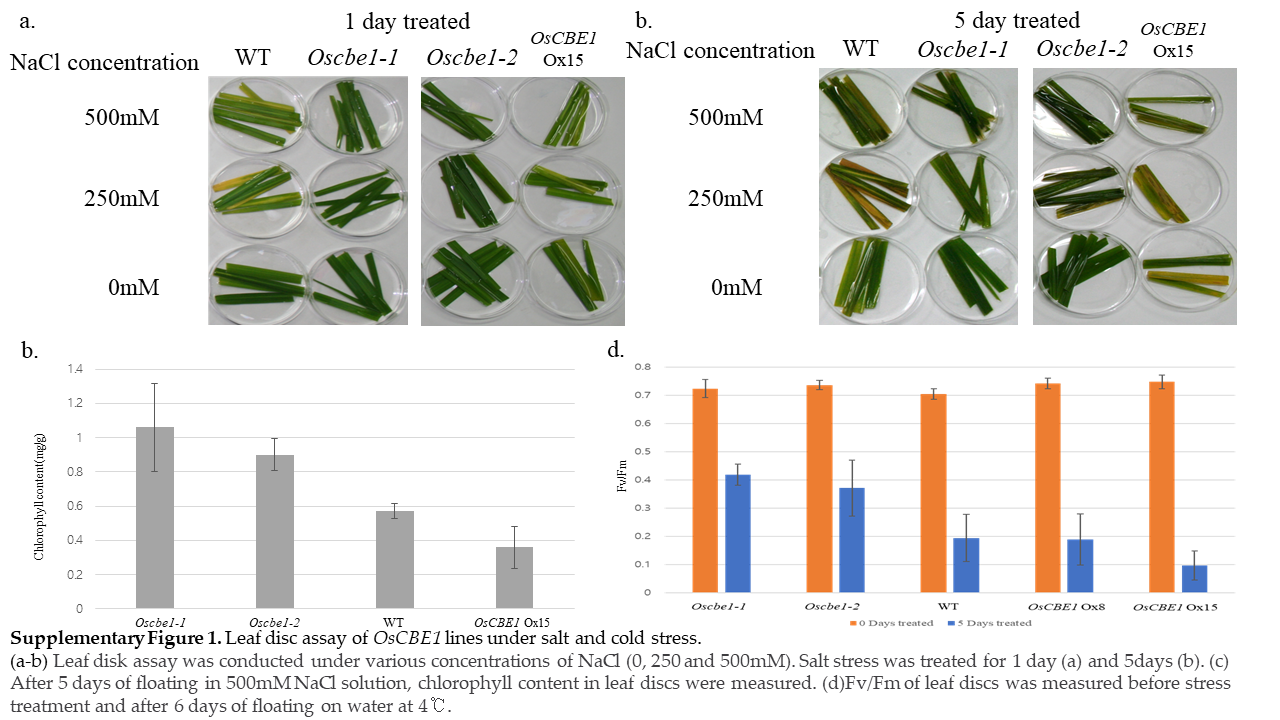

Supplement: Supplementary file 1 [file ijms-22-02487-s001.zip › 슬라이드9.TIF]
